# Multisystem regulation of reward and addiction: beyond the dopaminergic system

**DOI:** 10.3389/fphar.2026.1840096

**Published:** 2026-05-20

**Authors:** Oscar Arias-Carrión, Emmanuel Ortega-Robles, Mario Treviño, Magdalena Guerra-Crespo, Elias Manjarrez, Ernst Pöppel

**Affiliations:** 1 División de Neurociencias Clínica, Instituto Nacional de Rehabilitación, Mexico City, Mexico; 2 Tecnologico de Monterrey, Escuela de Medicina y Ciencias de la Salud, Mexico City, Mexico; 3 Laboratorio de Plasticidad Cortical y Aprendizaje Perceptual, Instituto de Neurociencias, Universidad de Guadalajara, Guadalajara, Mexico; 4 Laboratory of Regenerative Medicine, Department of Physiology, Faculty of Medicine, National Autonomous University of Mexico, Mexico City, Mexico; 5 Instituto de Fisiología, Benemérita Universidad Autónoma de Puebla, Puebla, Mexico; 6 Institute of Medical Psychology, Ludwig Maximilian University Munich, Munich, Germany

**Keywords:** addiction vulnerability, dopamine, mesolimbic system, reinforcement learning, reward system, synaptic plasticity, valence systems

## Abstract

Addiction to illicit drugs remains a major global health challenge that requires integrative efforts across neuroscience, psychiatry, and pharmacology. A deeper understanding of the neural mechanisms underlying addictive behavior is essential for developing effective and targeted interventions. It is increasingly recognized that addiction cannot be fully explained by dysfunction within dopaminergic circuits alone but rather reflects maladaptive interactions across distributed neuromodulatory and glial networks. Emerging evidence suggests that reward and aversion are better conceptualized within a unified framework of valence processing, in which dopaminergic activity dynamically interacts with orexinergic, histaminergic, endocannabinoid, metabolic, and stress-related systems to shape motivation, reinforcement learning, and affective regulation. Within this expanded architecture, the subventricular tegmental nucleus (SVTg) has recently been identified as a novel brainstem node that may regulate dopaminergic excitability and integrate signals from cortical, limbic, and stress-related circuits. This review synthesizes converging molecular, circuit, and translational evidence supporting a multisystem, network-based model of reward regulation, emphasizing how dysregulation across these systems contributes to addiction vulnerability and related psychiatric phenotypes characterized by compulsivity and impaired valence regulation. Therapeutic advances, including SVTg-targeted neuromodulation, orexin receptor antagonists, histaminergic modulation, and glucagon-like peptide-1 (GLP-1)–based interventions, illustrate the translational potential of this distributed perspective. We argue that future progress will depend on integrating single-cell transcriptomics, real-time neuroimaging, computational psychiatry, and pharmacogenomics to develop mechanistically informed, personalized treatments. Reconceptualizing reward and addiction as emergent properties of distributed brainstem–cortical circuits offers a transformative path toward precision medicine in substance use and related neuropsychiatric disorders.

## 1 Introduction

Valence processing—the capacity to assign positive or negative value to internal or external stimuli—is fundamental to survival. It allows organisms to approach rewarding contexts and avoid harmful ones, integrating sensory, motivational, and emotional information across multiple neural systems (Hu, 2016; Mondoloni et al., 2022). Within this framework, reward processing represents one dimension of valence and is supported by dopaminergic, glutamatergic, GABAergic, peptidergic, and other neuromodulatory systems that regulate affect and learning through distributed cortico–subcortical circuits involved in reward, stress, and executive control (Hu, 2016; Mondoloni et al., 2022; Jones et al., 2024; Brida and Day, 2025). These circuits are highly adaptive but also vulnerable to long-lasting plastic changes, and their dysregulation is thought to underlie susceptibility to substance use disorders and related neuropsychiatric conditions (Jones et al., 2024; Brida and Day, 2025).

**FIGURE 1:**
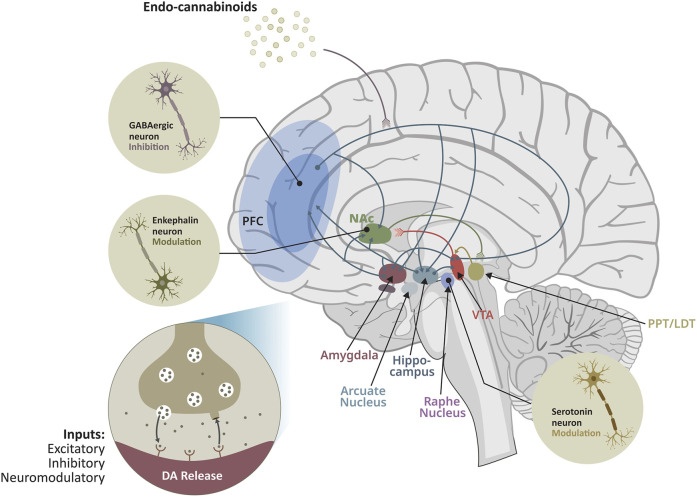
Distributed brainstem–cortical neuromodulatory networks regulating dopamine release and motivational salience. The mesocorticolimbic system orchestrates motivation, reinforcement learning, and valence allocation through highly conserved neuromodulatory interactions. Dopaminergic neurons in the ventral tegmental area (VTA) integrate excitatory, inhibitory, and neuromodulatory inputs originating from the prefrontal cortex (PFC), nucleus accumbens (NAc), hippocampus, amygdala, dorsal raphe nucleus, arcuate nucleus, and pedunculopontine/laterodorsal tegmental complex (PPT/LDT). These long-range projections converge onto dopamine terminals to regulate phasic and tonic dopamine release, thereby dynamically shaping motivational drive and reward prediction error signaling. GABAergic and enkephalinergic striatal populations exert essential inhibitory and modulatory control over the gain of dopamine neuron activity, while serotonergic projections from the raphe nuclei further tune behavioral flexibility, affective state, and impulse control. Endocannabinoids, released on demand, provide a retrograde signal that suppresses presynaptic inhibition and modulates glutamatergic and GABAergic input, thereby facilitating DA release under states of heightened salience or physiological need. This distributed architecture demonstrates that reward processing emerges not from isolated dopaminergic mechanisms but from coordinated interaction across multiple neuromodulatory systems that integrate internal state, context, and experience to bias action selection and addiction vulnerability.

Valence-guided behavior depends on the integration of both appetitive and aversive information within overlapping networks. Primary rewards (e.g., food, water) serve immediate physiological needs, whereas secondary rewards (e.g., novelty and social recognition) emerge through learned associations. Conversely, aversive signals, including punishment, threat, and loss, engage partially overlapping circuits that promote avoidance and inhibitory control. Rather than processing these signals in isolation, distributed neural systems flexibly encode both approach and avoidance in dynamic environments, linking basic reinforcement mechanisms to clinically relevant phenomena such as anhedonia and relapse (Figure 1).

For decades, the mesolimbic dopaminergic system has been regarded as the principal substrate of reinforcement learning and reward processing (Arias-Carrion and Poppel, 2007; Arias-Carrion et al., 2010; Brocka et al., 2018). However, it is now understood that dopamine (DA) also contributes to a much wider range of functions, including movement, attention, motivation, and the processing of aversive events (Glimcher, 2011; Fitzgerald and Day, 2025). Dopaminergic neurons in the ventral tegmental area (VTA) influence goal-directed behavior through projections to the nucleus accumbens (NAc), the prefrontal cortex (PFC), the amygdala, and the hippocampus (Kleinman and Foster, 2025). Traditionally, these neurons were conceptualized as reward prediction error encoders, signaling the discrepancy between expected and actual outcomes to update value representations (Schultz, 2013; Watabe-Uchida et al., 2017; Diederen and Fletcher, 2020; Cone et al., 2024; Kahnt and Schoenbaum, 2025). Nevertheless, growing evidence indicates that DA neurons are more heterogeneous, some responding to reward, others to aversion, and others to salience irrespective of valence, suggesting that DA codes not merely for reward but for motivational significance more generally (Lammel et al., 2014; Yuan et al., 2019; Bouchard et al., 2025). In line with incentive-sensitization accounts, mesolimbic dopamine is now thought to contribute primarily to incentive motivation, or “wanting,” rather than to the hedonic “liking” of rewards (Robinson and Berridge, 2025).

Recent work in mammals and invertebrates supports the view that valence processing emerges from multisystem interactions rather than from dopamine alone. Studies across species show that dopaminergic, cholinergic, and other signals can jointly encode both reward and punishment, including circuit motifs in which the omission of punishment is recoded as reward (McCurdy et al., 2021; Jovanoski et al., 2023). Such findings support the notion that reinforcement learning, motivation, and decision-making are dynamically integrated through neuromodulatory convergence. One of the most compelling recent advances has been the identification of the subventricular tegmental nucleus (SVTg), a previously overlooked brainstem structure that has been shown to exert inhibitory control over the lateral habenula, and to modulate dopaminergic tone, and thereby potentially shaping reward and anxiety states (Zichó et al., 2025). Rather than serving as a purely “reward center,” the SVTg has been proposed to operate as a valence node, balancing approach and avoidance signals within the lateral habenula (LHb)–ventral tegmental area axis (Chen and Hu, 2025). Optogenetic manipulations of this nucleus have been shown to bidirectionally influence reward seeking and aversion, suggesting that it may represent an evolutionarily conserved node within the distributed circuitry of valence regulation (Grillner, 2025; Zichó et al., 2025).

Together, these findings support a framework in which dopaminergic signaling participates in positive and negative motivational states within a broader, multisystem network of valence processing. From this perspective, addiction can be viewed as a maladaptive reorganization of distributed neuromodulatory circuits—including mesolimbic, stress, and executive-control networks—in which the balance between reward and aversion signaling is persistently disrupted. Existing accounts have predominantly focused on dopamine, stress, or specific neuromodulators in isolation, leaving an integrative, valence-based model of multisystem regulation underdeveloped. In this review, we synthesize molecular, circuit, genetic, and translational evidence into a distributed brainstem–cortical model of valence regulation, and link these mechanisms to addiction vulnerability, relapse, and emerging multi-target interventions. Our goal is to provide a field-shaping framework for precision psychiatry in substance use and related neuropsychiatric disorders.

## 2 Dopaminergic circuitry in valence processing and addiction

Dopaminergic neurons constitute a small yet highly specialized population distributed across the mesencephalon, diencephalon, and olfactory bulb, with the majority located in the ventral midbrain. Despite representing a minute fraction of brain neurons, DA cells exert disproportionate influence over movement, motivation, learning, and affect through extensive axonal arborization and widespread projections (Zimmerman and Grace, 2016; Rincón-Cortés and Grace, 2020; Haim et al., 2025). DA signaling contributes to a broader motivational framework encompassing both positive and negative valence. This functional diversity reflects heterogeneity in projection targets, electrophysiological properties, molecular identity, and co-transmission, as subsets of VTA neurons co-release GABA or glutamate, enabling bidirectional modulation of motivational state (Morales and Margolis, 2017).

The classical view of dopamine as a unitary “reward signal” has therefore been replaced by a distributed, valence-sensitive framework. The mesocorticolimbic system, linking the VTA with the NAc, amygdala, hippocampus, and PFC, is now understood as a network supporting reinforcement learning, incentive salience, and behavioral selection rather than a linear reward pathway (Li et al., 2021; Mondoloni et al., 2022). Within this system, distinct DA neuron populations increase firing in response to appetitive cues, decrease activity during aversive events, or encode motivational salience irrespective of valence (Lammel et al., 2014; Yuan et al., 2019; Bouchard et al., 2025). Consistent with incentive-sensitization theory, mesolimbic dopamine primarily amplifies incentive motivation (“wanting”) rather than hedonic pleasure (“liking”) (Robinson and Berridge, 2025). Experimental studies further demonstrate that mesolimbic DA activity sustains cue-triggered drug seeking and relapse following abstinence, highlighting its role in the persistence and generalization of maladaptive motivational states (Mo et al., 2025; Weber et al., 2025).

Dopaminergic function is not confined to ventral striatal circuits. The nigrostriatal pathway, originating in the substantia nigra pars compacta and projecting to the dorsal striatum, is classically associated with motor control but also contributes to effort allocation, action vigor, and motivational drive (Russo and Nestler, 2013; Obeso et al., 2017; Volkow and Blanco, 2023). Recruitment of dorsal striatal circuits is increasingly implicated in the transition from goal-directed drug use to habitual and compulsive behavior, linking dopaminergic signaling across motor, cognitive, and motivational domains (Lüscher and Janak, 2021).

Although DA neurons account for less than 0.01% of all brain neurons, their extensive axonal arborization amplifies their influence. A single midbrain DA neuron may form hundreds of thousands of synapses throughout the striatum and cortex, allowing local fluctuations in dopaminergic activity to propagate across large-scale networks. This architecture contributes to widespread effects on motivation, affect, and addiction-related processes.

At the molecular level, DA transmission operates through five G protein-coupled receptor subtypes grouped into D1-like (D1, D5) and D2-like (D2, D3, D4) families with complementary anatomical distributions and signaling properties (Matsuda et al., 2009; Beaulieu and Gainetdinov, 2011; Gerfen and Surmeier, 2011). These receptor systems interact to integrate reward- and punishment-related information during value-based decision-making (Soares-Cunha et al., 2016). Dopamine operates largely through volume transmission, allowing phasic and tonic signals to modulate neural populations over extended spatial and temporal scales (Rice and Cragg, 2008). Phasic burst firing encodes reward prediction errors that update value representations (Steinberg et al., 2013; Schultz, 2016), while other DA subpopulations encode aversive prediction errors or threat-related salience (Lammel et al., 2014; de Jong et al., 2019). These observations position dopamine as a multidimensional modulator of motivational significance.

Drugs of abuse exploit this architecture by producing exaggerated dopaminergic responses in mesolimbic circuits, thereby hijacking adaptive learning mechanisms and assigning excessive incentive salience to drug-associated cues (Koob and Volkow, 2016). Repeated exposure induces synaptic plasticity within the VTA and NAc, weakens inhibitory control over DA neurons, and stabilizes maladaptive learning through neurotrophic and gene-regulatory mechanisms (Lüscher and Malenka, 2011; Tan et al., 2012; Eshel et al., 2015; Pascoli et al., 2018; Nestler, 2025). Paradoxically, chronic addiction is characterized by reduced striatal DA release and lower D2 receptor availability, correlating with anhedonia and diminished sensitivity to natural rewards (Volkow et al., 2017; Volkow et al., 2019). Thus, addiction cannot be explained as a purely hyperdopaminergic state but instead reflects a dysregulation of valence and motivational processing, combining hypersensitivity to drug cues with blunted responsiveness to non-drug rewards and aversive signals.

These observations highlight the limitations of strictly dopamine-centric models and motivate the need for integrative frameworks incorporating distributed neuromodulatory interactions. Emerging therapeutic strategies increasingly target such interactions, including metabolic modulators such as glucagon-like peptide-1 (GLP-1) receptor agonists, which may indirectly recalibrate dopaminergic function and motivational priorities (Egecioglu et al., 2013; Skibicka, 2013). In the following sections, we build on this foundation to examine how non-dopaminergic systems and brainstem–cortical circuits jointly regulate valence and contribute to addiction vulnerability.

## 3 Drug-induced neuroadaptations and the processing of natural rewards

Addiction cannot be understood as a mere amplification of normal reward processing. Instead, addictive substances selectively recruit and distort neural circuits that evolved to encode motivational valence, biasing the balance between reward, aversion, and stress. Although both natural and drug rewards engage overlapping nodes within mesocorticolimbic circuits, they induce qualitatively distinct forms of synaptic plasticity with divergent behavioral consequences. Under these conditions, repeated drug exposure gives rise to persistent and often compensatory changes in neural circuits—here referred to as neuroadaptations—which represent a subset of neuroplastic processes and are associated with allostatic adjustments that progressively alter reward processing and motivational control. Natural rewards, such as food, novelty, and social interaction, elicit transient, homeostatically regulated dopaminergic responses that support flexible reinforcement learning and adaptive decision-making, whereas drug-induced neuroadaptations contribute to reduced sensitivity to natural rewards and a shift toward drug-centered valuation. In this context, dopamine encodes prediction errors that support adaptive motivational balance.

In contrast, addictive drugs produce supraphysiological and temporally prolonged dopaminergic elevations that overwhelm regulatory mechanisms and induce maladaptive synaptic and transcriptional changes, progressively eroding behavioral flexibility and favoring compulsive drug seeking (Koob, 2021; Volkow and Blanco, 2023). Natural and drug reinforcers compete for shared substrates within valence-processing networks, particularly in the NAc and orbitofrontal cortex. With repeated drug exposure, valuation mechanisms become increasingly biased toward drug-associated cues, while the motivational impact of natural rewards diminishes, a hallmark prediction of incentive-sensitization theory (Robinson and Berridge, 2025). This leads to a narrowing of motivational focus, with drug-seeking persisting despite negative consequences (Hyman et al., 2006; Robinson and Berridge, 2025).

Crucially, this reweighting of motivational priorities is reinforced by recruitment of stress and aversion systems. Chronic drug exposure enhances corticotropin-releasing factor (CRF) signaling and activity within the extended amygdala, generating dysphoria, anxiety, and irritability that further suppress the reinforcing value of natural rewards and promote relapse through negative reinforcement (Koob et al., 2020). At the synaptic level, drug-induced neuroadaptations include increased AMPA/NMDA receptor ratios, dendritic spine remodeling in the NAc and PFC, and disrupted corticostriatal connectivity, collectively weakening top-down control while strengthening cue-driven and habit-like responses (Lüscher and Janak, 2021; Nestler, 2025). These changes are stabilized by epigenetic mechanisms, including ΔFosB accumulation and chromatin remodeling, which sustain maladaptive transcriptional programs long after drug cessation (Maze and Nestler, 2011; Nestler, 2025).

Dopamine dysregulation represents only one component of a broader multisystem pathology. Glutamatergic, GABAergic, orexinergic, and glial mechanisms critically shape how reward, aversion, and stress signals are integrated, while neuroimaging studies demonstrate exaggerated responses to drug cues, blunted activation to natural rewards, and reduced prefrontal engagement during value-based decisions (Trifilieff and Martinez, 2014; Skupio et al., 2020; James and Aston-Jones, 2022). These findings support a network-level view of valence dysregulation in addiction. Clinically, this perspective motivates interventions aimed at restoring the salience of natural reinforcers and rebalancing reward–aversion networks, including pharmacological and neuromodulatory approaches targeting glutamate, orexin, and stress-related circuits (Eren-Yazicioglu et al., 2021; Klausen et al., 2022).

## 4 Neuroplasticity and the transition to compulsive drug-seeking behavior

Repeated exposure to addictive substances induces progressive neuroplastic changes across reward, stress, and control circuits, driving the shift from voluntary use to compulsive drug seeking despite adverse consequences. These changes reflect broader neuroplastic processes—understood here as activity-dependent modifications in synaptic, cellular, and circuit-level organization that support learning and behavioral adaptation. In the context of addiction, the neuroadaptations described above represent specific, often maladaptive forms of neuroplasticity that progressively bias reinforcement learning and action selection toward compulsive drug-seeking behavior. This transition reflects maladaptive reinforcement learning in which drug-associated cues acquire excessive motivational salience at the expense of natural rewards. Within the incentive-sensitization framework, repeated drug exposure sensitizes mesolimbic dopamine signaling—particularly within the NAc—amplifying the motivational impact of drug-paired stimuli (Volkow and Blanco, 2023; Robinson and Berridge, 2025). Although sensitization can occur with natural reinforcers, addiction represents a persistent and biased state in which these adaptations become entrenched and dominate behavioral control.

Beyond dopamine sensitization, affective dysregulation is a key driver of compulsion. The opponent-process and allostatic models converge in proposing that chronic drug use induces within-system adaptations that blunt mesocorticolimbic responses to natural rewards and between-system adaptations that recruit stress circuitry. In particular, persistent hyperactivity of CRF signaling during withdrawal imposes an allostatic load that promotes dysphoria, craving, and relapse through negative reinforcement mechanisms (Koob et al., 2020). Reward deficiency and stress excess interact to maintain compulsive use.

At the synaptic level, the transition to compulsive drug seeking is driven not only by the presence of drug-induced neuroadaptations but by their progressive stabilization and functional integration within corticostriatal circuits. Repeated drug exposure biases synaptic plasticity mechanisms toward the strengthening of drug-associated inputs, including persistent potentiation of glutamatergic transmission and impaired capacity for synaptic depression. These processes promote the preferential encoding of drug-related cues and reduce behavioral flexibility. In parallel, dopamine-dependent modulation of synaptic plasticity further amplifies the salience of drug-paired stimuli, facilitating the shift from goal-directed to habitual and ultimately compulsive responding (Hyman et al., 2006; Lüscher and Malenka, 2011; Wolf, 2016). These synaptic changes, while reflecting drug-induced neuroadaptations, are instantiated through broader neuroplastic mechanisms that govern reinforcement learning and action selection, progressively biasing behavior toward compulsive drug seeking.

**FIGURE 2:**
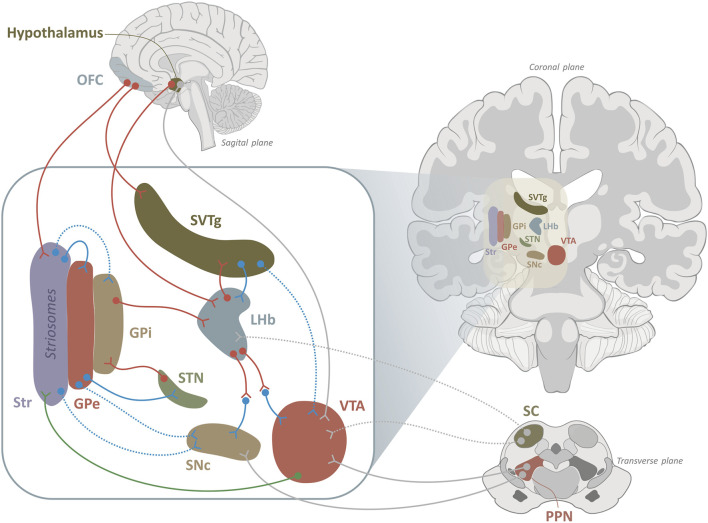
Cortico–basal ganglia–habenular–midbrain circuitry shaping dopaminergic valence signaling. This schematic depicts convergent cortical, basal ganglia, hypothalamic, and brainstem pathways regulating motivational valence and midbrain dopamine output. The orbitofrontal cortex (OFC) provides top-down evaluative signals to striatum (Str; including striosomal compartments) and directly to the subventricular tegmental nucleus (SVTg), linking outcome valuation to reward- and aversion-related circuits. Within the basal ganglia, GABAergic Str projections engage the canonical indirect pathway (Str → external globus pallidus [GPe] → subthalamic nucleus [STN] → internal globus pallidus [GPi]), shaping excitatory GPi output to the lateral habenula, a key node for negative prediction-error and aversive signaling. The LHb integrates excitatory and inhibitory inputs from basal ganglia, hypothalamic, and brainstem sources. Hypothalamic afferents convey homeostatic and interoceptive state information, while the SVTg forms a reciprocal loop with the LHb, enabling bidirectional modulation of habenular excitability. LHb output influences dopaminergic neurons in the ventral tegmental area (VTA) and substantia nigra pars compacta (SNc) predominantly via GABAergic relay neurons, suppressing dopamine activity during aversive or worse-than-expected outcomes. In parallel, the pedunculopontine nucleus (PPN) provides direct modulatory input to VTA and SNc, linking arousal and salience to dopaminergic firing, while superior colliculus (SC) inputs represent putative pathways conveying rapid sensory and orienting signals. Dopaminergic projections from the VTA to the striatum complete a feedback loop supporting reinforcement learning and adaptive updating of action–outcome contingencies. Together, this distributed network enables OFC-driven valuation and state-dependent signals to dynamically bias approach versus avoidance behavior by regulating the timing and magnitude of dopaminergic responses. Line type denotes strength of evidence (solid, supported and central; dashed, putative or modulatory). Line color indicates predominant neurotransmitter identity: blue, GABAergic; red, glutamatergic; green, dopaminergic; grey, mixed or unspecified (additional modulatory input).

At the circuit level, behavioral control progressively shifts from ventral striatal and prefrontal regions supporting goal-directed action toward dorsal striatal circuits associated with habitual responding (Figure 2). This corticostriatal remodeling weakens executive regulation and favors rigid stimulus–response patterns (Belin et al., 2013; Everitt and Robbins, 2016; Marti-Prats et al., 2023). Importantly, dorsal striatal engagement does not merely reflect habit formation but the persistence of reinforcement learning biased toward drug-related stimuli. Within this framework, striosomal compartments provide inhibitory feedback to midbrain dopamine neurons, fine-tuning reinforcement sensitivity and stabilizing maladaptive action policies, thereby contributing to the inflexibility characteristic of compulsive drug use (Gerfen and Surmeier, 2011; Graybiel and Matsushima, 2023).

Emerging perspectives further emphasize distortions in reward anticipation and prediction-error signaling. Drug-evoked dopamine surges act as exaggerated prediction-error–like signals that strengthen maladaptive cue–drug associations, while chronic exposure blunts responses to natural reinforcers (Hyman et al., 2006; Volkow et al., 2019). These processes consolidate perseverative seeking despite explicit awareness of harm.

Individual vulnerability is shaped by genetic and epigenetic factors, as well as by non-dopaminergic modulators of arousal and stress. The orexin–hypocretin system, for example, amplifies cue- and stress-induced reinstatement via projections to mesolimbic and stress circuits, motivating exploration of orexin receptor antagonists as relapse-reducing interventions (James and Aston-Jones, 2022; Nestler, 2025). Collectively, these findings indicate that compulsive drug seeking emerges from convergent plasticity across reward, aversion, and control systems, reinforcing the need for multisystem, circuit-informed therapeutic strategies.

## 5 Dopaminergic dysregulation in addiction

The reinforcing properties of addictive substances largely derive from their capacity to enhance dopaminergic signaling within mesocorticolimbic circuits, particularly the NAc. Despite pharmacological heterogeneity, most drugs of abuse converge on this pathway to amplify dopamine transmission and reinforce drug-seeking behavior. Psychostimulants exemplify this effect by producing rapid and supraphysiological dopamine elevations through blockade or reversal of dopamine transporters, thereby strengthening maladaptive reinforcement learning and biasing behavior toward compulsive consumption (Volkow and Blanco, 2023). Other drug classes act indirectly: alcohol and opioids disinhibit VTA dopamine neurons via GABAergic mechanisms, while nicotine directly excites mesolimbic dopamine neurons through nicotinic receptors. Across drug classes, disruption of dopamine signaling within the NAc reliably impairs self-administration, underscoring its central role in maintaining drug-seeking behavior (Koob and Volkow, 2016; Koob, 2021).

However, addiction cannot be explained by dopaminergic mechanisms alone. Chronic drug exposure induces persistent adaptations in dopaminergic signaling, including altered firing patterns of VTA neurons, changes in dopamine synthesis and receptor regulation, and downstream modifications in intracellular cascades. These adaptations are stabilized by coordinated changes in glutamatergic and GABAergic transmission, reflecting dysregulation across interacting neurotransmitter systems (Lüscher and Janak, 2021). At the molecular level, transcriptional regulators such as ΔFosB accumulate in the NAc and, together with epigenetic mechanisms including histone modification and DNA methylation, consolidate maladaptive plasticity and confer long-lasting relapse vulnerability (Nestler, 2025). The principal neurotransmitter systems that modulate dopaminergic function and contribute to reward dysregulation in addiction are summarized in Table 1.

**TABLE 1:** Neurotransmitter systems modulating reward and addiction.

System	Mechanism of action in reward circuits
Dopamine (DA)	Encodes reward prediction errors through phasic burst firing in the ventral tegmental area, driving reinforcement learning, motivational salience, and goal-directed behavior. Dysregulated DA transmission underlies maladaptive plasticity in addiction (Koob and Volkow, 2016; Schultz, 2016)
GABA	Local interneurons in the VTA and inputs from the basal ganglia inhibit DA neuron excitability. Chronic drug exposure reduces GABAergic control, amplifying cue reactivity and promoting compulsive drug seeking (Tan et al., 2012; Eshel et al., 2015)
Glutamate (Glu)	Provides excitatory drive to DA neurons in the VTA and nucleus accumbens. Synaptic potentiation, mediated by altered AMPA/NMDA receptor ratios, consolidates drug-associated memories; endocannabinoids regulate glutamate release to shape plasticity (Lüscher and Malenka, 2011; Morales and Margolis, 2017)
Serotonin (5-HT)	Modulates DA neuron firing and regulates reward salience, mood, and aversive learning. Dysregulated 5-HT signaling contributes to impulsivity and altered reinforcement valuation (Cools et al., 2011; Volkow and Boyle, 2018)
Acetylcholine (ACh)	Cholinergic inputs from the pedunculopontine and laterodorsal tegmental nuclei facilitate DA burst firing and support striatal synaptic plasticity, enhancing cue–reward associations (Mahler et al., 2014b; Morales and Margolis, 2017)
Orexin/Hypocretin	Links arousal to reward processing through projections to the VTA and nucleus accumbens. Promotes reward-seeking and mediates stress-induced relapse; orexin-1 receptor blockade reduces reinstatement (Mahler et al., 2014a; James and Aston-Jones, 2022)
Endocannabinoids	Retrograde messengers that regulate presynaptic glutamate and GABA release, thereby modulating DA transmission. Δ9-tetrahydrocannabinol (THC) enhances mesolimbic DA release and alters long-term reward sensitivity (Katona and Freund, 2012; Volkow et al., 2017)
Enkephalins	Opioid peptides that inhibit aversive signaling and enhance DA activity via disinhibition of VTA neurons. Opioid–dopamine interactions reinforce drug reward and promote compulsive use (Bruchas et al., 2010; Koob and Volkow, 2016)
Histamine	Exerts receptor-specific modulation of DA release: H_1_ receptor activation facilitates mesolimbic excitability, whereas H_3_ receptor signaling suppresses DA output and cognitive flexibility. H_3_ antagonists reduce relapse vulnerability and compulsive behaviors (Ligneau et al., 2007; Panula and Nuutinen, 2013)

Dopaminergic dysregulation is accompanied by impaired executive control. Chronic substance use disrupts prefrontal–striatal–thalamic circuits critical for decision-making and inhibitory regulation. Neuroimaging studies consistently demonstrate hypoactivity and structural alterations in the dorsolateral PFC, orbitofrontal cortex, and anterior cingulate cortex, correlating with impulsivity, reduced cognitive flexibility, and heightened cue reactivity across substance use disorders (Volkow and Boyle, 2018; Zilverstand et al., 2018). Within the NAc, regional heterogeneity further shapes reinforcement: the shell subregion integrates appetitive and aversive signals, and cue-evoked dopamine release in this region encodes motivational predictions rather than reward *per se*, reinforcing drug-oriented behavior (Mohebi et al., 2019).

From a systems perspective, addiction reflects a maladaptive reorganization of reward and stress networks. Allostatic models propose that chronic drug use induces both reduced dopaminergic function and heightened recruitment of stress-responsive circuits, including the hypothalamic–pituitary–adrenal axis and CRF pathways, promoting dysphoria and relapse during withdrawal (Koob, 2021). Individual susceptibility is further shaped by genetic and epigenetic variability. Emerging interventions, such as GLP-1 receptor agonists, highlight the translational potential of targeting dopamine within broader metabolic and glutamatergic networks, offering a shift toward precision strategies that restore motivational balance and reduce relapse risk (Eren-Yazicioglu et al., 2021; Klausen et al., 2022).

## 6 Dynorphin system modulation of dopamine signaling

The dynorphin/kappa-opioid receptor (KOR) system is a major regulator of negative valence within mesolimbic circuits and plays a central role in stress responsivity, dysphoria, and relapse vulnerability in addiction. KORs are Gi/o-coupled receptors whose activation broadly suppresses neuronal excitability and neurotransmitter release, while engaging intracellular signaling cascades that influence gene transcription and synaptic plasticity (Bruchas et al., 2010; Tejeda and Bonci, 2019). Dynorphin/KOR signaling primarily encodes aversive motivational states and constrains dopaminergic output.

Activation of KORs by dynorphin suppresses dopamine release in the VTA and NAc, producing dysphoria and aversion that are prominent during withdrawal and stress. This inhibition arises through combined presynaptic suppression of DA terminals and modulation of glutamatergic and GABAergic inputs to DA neurons. Functionally, these effects bias motivation toward negative reinforcement, promoting drug seeking as a means of alleviating aversive internal states. Preclinical studies show that KOR activation engages p38 MAPK–dependent mechanisms within mesolimbic circuits, reinforcing depressive-like states, whereas KOR antagonism reverses stress-induced anhedonia and attenuates stress-triggered reinstatement of drug seeking (Carlezon and Krystal, 2016).

Chronic drug exposure induces enduring adaptations within the dynorphin/KOR system. While early drug exposure is dominated by enhanced dopaminergic signaling, prolonged use leads to compensatory upregulation of prodynorphin expression and KOR function within striatal and limbic regions. This overcorrection establishes an allostatic state characterized by reduced sensitivity to natural rewards and heightened negative affect, increasing reliance on drug intake for relief. Through this mechanism, dynorphin/KOR signaling contributes to the transition from positively reinforced use to compulsive drug seeking driven by negative reinforcement (Koob and Volkow, 2016).

Although KOR signaling is temporally dynamic, its most robust behavioral consequence is sustained suppression of phasic DA signaling and distortion of reward prediction error processing, sufficient to blunt responses to natural rewards and bias learning toward rigid, drug-centered motivational strategies (Ehrich et al., 2014; Al-Hasani et al., 2015; Ehrich et al., 2015; Tejeda and Bonci, 2019). KORs also interact with glutamatergic, GABAergic, and noradrenergic pathways within the amygdala, bed nucleus of the stria terminalis, and locus coeruleus, and with CRF signaling in the extended amygdala to amplify stress-related responses (Land et al., 2008; Crowley and Kash, 2015; Valentino and Van Bockstaele, 2015). This positions dynorphin/KOR signaling as an interface between stress and reward systems.

Therapeutically, targeting the dynorphin/KOR axis represents a promising strategy for restoring balance of valence. Long-acting and biased KOR antagonists reduce stress-induced relapse-like behavior and alleviate anxiety- and depressive-like states in preclinical models without intrinsic reinforcing effects (Carlezon and Krystal, 2016; Tejeda and Bonci, 2019; Valentino et al., 2024). Modulation of dynorphin/KOR signaling may attenuate negative affect and rebalance motivational processing in addiction.

## 7 Genetic and neurobiological variability in addiction and aversion susceptibility

Vulnerability to addiction and maladaptive avoidance reflects interindividual variability in how neural systems encode and regulate motivational valence. Genetic liability, neurobiological architecture, and environmental exposure jointly shape sensitivity to both rewarding and aversive outcomes, influencing not only compulsive drug seeking but also stress reactivity and withdrawal severity. Twin and genome-wide association studies estimate that heritability accounts for approximately 40%–60% of the risk for alcohol use disorder, with comparable but more variable estimates reported across other substance use disorders (Verhulst et al., 2015; Zhou et al., 2020; Deak and Johnson, 2021; Miller et al., 2024). Importantly, this genetic liability appears partly domain-general, consistent with evidence for a shared latent addiction-risk factor spanning alcohol, tobacco, cannabis, and opioid use disorders (Hatoum et al., 2022). This shared vulnerability is thought to reflect core traits—including impulsivity, stress sensitivity, and reduced executive control—that bias motivational processing before substantial drug exposure (Kozak et al., 2019; Miller et al., 2024).

One of the most consistently replicated neurobiological correlates of vulnerability is reduced dopamine D2 receptor availability within striatal circuits. Lower D2/3 receptor density in the dorsal striatum and NAc is associated with diminished inhibitory control, blunted reward sensitivity, and increased risk-taking behavior (Bocarsly et al., 2019; Volkow et al., 2019; Jaworska et al., 2020). Longitudinal imaging studies suggest that, in at least a subset of individuals, reduced baseline D2 receptor availability precedes escalation of drug use and predicts craving intensity, supporting a hypodopaminergic endophenotype characterized by anhedonia and compulsivity (Parvaz et al., 2011; Jaworska et al., 2020). This phenotype interacts with genetic variation across dopamine, glutamate, serotonin, and opioid systems to shape individual trajectories of reward and aversion sensitivity (Polimanti et al., 2020; Speranza et al., 2021).

Epigenetic mechanisms provide a critical bridge between environmental exposure and persistent vulnerability. Stress and early-life adversity induce durable DNA methylation and histone modifications within prefrontal–striatal and amygdalar circuits, biasing motivational drive, impulse control, and emotional learning (Nestler, 2014; Cadet and Jayanthi, 2021). These epigenetic signatures converge on networks governing reinforcement learning and threat appraisal, linking environmental stressors to long-term dysregulation of motivational processing (Zillich et al., 2022).

Genetic overlap between addiction and major psychiatric disorders—including depression, schizophrenia, and obsessive–compulsive disorder—further supports a shared polygenic architecture of motivational dysregulation (Polimanti et al., 2020). Variants affecting monoaminergic transmission and stress-regulatory pathways (e.g., COMT, MAOA, CRHR1, FKBP5) jointly influence reward responsiveness and aversion sensitivity, generating distinct affective vulnerability profiles (Anderson et al., 2019; Molins et al., 2022). At the circuit level, converging evidence implicates dysfunction of prefrontal–striatal control loops, in interaction with striatal cue reactivity and amygdalar stress responses, as a central mechanism underlying impaired motivational flexibility (Hu et al., 2015; Zilverstand et al., 2018). Key mechanisms contributing to individual vulnerability are summarized in Table 2.

**TABLE 2:** Multilevel determinants of addiction vulnerability.

Mechanism/Risk factor		Effect on reward and addiction processing
Circuit-level mechanisms	SVTg–LHb–VTA microcircuit	The subventricular tegmental nucleus (SVTg) has been proposed to exert bidirectional control over dopaminergic excitability, in part by inhibiting the lateral habenula to reduce aversive signaling and facilitate persistent reward pursuit. Dysregulation of this pathway may bias reinforcement toward compulsive behaviors (Jovanoski et al., 2023; Grillner, 2025; Zichó et al., 2025)
	Orexin–CRF interaction	Orexinergic projections to the VTA couple arousal with motivational drive, while corticotropin-releasing factor (CRF) enhances stress-induced relapse. Their convergence amplifies vulnerability to reinstatement of drug seeking (Mahler et al., 2014a; Koob and Schulkin, 2019; James and Aston-Jones, 2022)
	GABAergic disinhibition	Chronic drug use diminishes inhibitory GABAergic control over VTA dopamine neurons, amplifying reward responses and enhancing cue salience (Tan et al., 2012; Eshel et al., 2015)
	Striatal plasticity	Repeated drug exposure remodels corticostriatal connectivity and strengthens AMPA/NMDA signaling in the nucleus accumbens. This shift promotes habitual responding and reduces prefrontal control over behavior (Lüscher and Malenka, 2011; Everitt and Robbins, 2016; Marti-Prats et al., 2023)
Molecular/synaptic-level mechanisms	Cannabinoid regulation	Endocannabinoid signaling modulates presynaptic glutamate and GABA release, enhancing mesolimbic dopamine transmission. Δ9-tetrahydrocannabinol (THC) alters reward salience and cue reactivity, especially during sensitive developmental periods (Katona and Freund, 2012; Volkow et al., 2017; Zimmermann et al., 2019)
	Histaminergic control	Histamine regulates mesolimbic excitability in a receptor-specific manner: H1 receptor activation facilitates reinforcement learning, whereas H_3_ antagonism restores dopaminergic tone and reduces compulsive drug seeking (Ligneau et al., 2007; Panula and Nuutinen, 2013)
Risk factors	Adolescent THC exposure	THC exposure during adolescence induces long-lasting transcriptomic alterations in the nucleus accumbens, heightens sensitivity to reward-predictive cues, and increases long-term addiction risk (Renard et al., 2017; Miller et al., 2019; Zuo et al., 2022; Murray et al., 2025)

From a translational perspective, this variability argues against uniform treatment approaches. Interventions targeting corticostriatal control and motivational balance—such as GLP-1 receptor agonists and neuromodulatory techniques including repetitive transcranial magnetic stimulation (rTMS), transcranial direct current stimulation (tDCS), and deep brain stimulation (DBS)—illustrate emerging strategies to address both reward hypo-reactivity and aversive hyper-responsiveness (Hanlon et al., 2015; Bari et al., 2018). Integrating genetic, epigenetic, and circuit-level markers will be essential for precision approaches aimed at improving treatment stratification and relapse prevention.

## 8 Hypocretin/orexin system as a modulator of motivational valence and relapse

The hypocretin/orexin (Hcrt) system is a central regulator of motivational intensity rather than a primary reward signal, linking arousal, interoceptive state, and stress to the allocation of effort toward salient outcomes. Originating from neurons in the lateral hypothalamus (LH), perifornical area (PeF), and dorsomedial hypothalamus (DMH), orexinergic projections innervate key arousal, stress, and reward-related regions, including the VTA, NAc, amygdala, and PFC. Through these connections, Hcrt signaling amplifies both appetitive and aversive motivational states and modulates vulnerability to substance use disorders (Mahler et al., 2014a; Tunisi et al., 2021; James and Aston-Jones, 2022).

At the molecular level, orexin receptors are G protein–coupled receptors whose signaling enhances neuronal excitability and synaptic plasticity in a context-dependent manner (Baimel and Borgland, 2017). Functionally, Hcrt signaling increases the gain of motivational circuits, particularly under conditions of high arousal, stress, or physiological need. Within the VTA, activation of orexin receptor 1 (OX1R) enhances dopamine neuron excitability and promotes burst firing, thereby increasing dopamine release in downstream targets and potentiating the motivational impact of reward-predictive cues, including drug-associated cues (Uramura et al., 2001; Baimel and Borgland, 2017). Consistent with this role, pharmacological or genetic disruption of Hcrt signaling reduces drug self-administration, conditioned place preference, and behavioral sensitization, whereas activation of the system promotes reinstatement of drug seeking (Baimel and Borgland, 2017; James and Aston-Jones, 2022; Thomas et al., 2022).

A major advance in the field is the recognition that the orexin system itself undergoes experience-dependent plasticity in addiction. Chronic exposure to highly salient rewards, including cocaine, opioids, and alcohol, produces a persistent increase in the number of orexin-immunoreactive neurons in the LH across species, including humans. The “orexin reserve” model proposes that a latent population of hypothalamic neurons can be recruited under sustained motivational demand. While this reserve supports adaptive behaviors under physiological conditions, chronic drug exposure persistently engages it, resulting in exaggerated orexin tone and a hypermotivated, drug-focused state (James and Aston-Jones, 2022). Baseline orexin neuron number predicts cocaine demand in rodents before addiction-like behavior, suggesting a contribution to trait vulnerability, although human data indicate that near-complete orexin loss, as in narcolepsy, does not confer clear protection against substance use (Dimitrova et al., 2011; Verstraete and Van Den Bossche, 2025). Nonetheless, preclinical models consistently show that orexin deficiency or receptor blockade attenuates withdrawal severity and relapse-like behavior (Georgescu et al., 2003; Quarta et al., 2010).

Functional specialization within the orexin system further refines its role in valence processing. LH orexin neurons preferentially encode reward prediction, high-effort seeking, and cue-induced relapse, whereas DMH and PeF populations are more strongly engaged by stress and arousal signals, consistent with their differential projections to reward- versus stress-related circuits (Mahler et al., 2014a; Baimel and Borgland, 2017; James and Aston-Jones, 2022). Through interactions with CRF and noradrenergic systems, orexin signaling integrates arousal and motivational state, amplifying both cue-driven craving and stress-related relapse (Mahler et al., 2014a; James and Aston-Jones, 2022; Verstraete and Van Den Bossche, 2025).

Therapeutically, the orexin system represents a compelling target for restoring motivational balance. Selective OX1R antagonists reduce drug demand and cue-induced reinstatement in preclinical models, often without intrinsic sedative or reinforcing effects. Clinically approved dual orexin antagonists, such as suvorexant, also reduce opioid seeking and relapse-like behavior in animal studies, supporting the translational relevance of targeting orexin-dependent motivational amplification (Kim et al., 2012; Illenberger et al., 2023). Orexin modulation may therefore dampen pathological motivational gain while preserving adaptive arousal and reward processing.

## 9 Corticotropin-releasing factor and orexin interactions in stress, valence, and addiction vulnerability

Interactions between the CRF and hypocretin/orexin systems constitute a central interface linking stress, arousal, and motivational drive. Stress-induced activation of CRF pathways increases the excitability of Hcrt neurons in the lateral hypothalamus and perifornical area, thereby potentiating dopaminergic and glutamatergic transmission in mesocorticolimbic circuits (Koob and Schulkin, 2019). This coupling links stress-related states with increased motivational drive underlying craving and relapse, helping to explain why stress is among the most powerful triggers of drug seeking.

CRF acts primarily through CRF_1_ and CRF_2_ receptors distributed across corticolimbic and extended-amygdala regions. Rather than detailing receptor-specific signaling cascades, a key functional distinction is that CRF_1_-dominated pathways enhance stress-related excitability and aversive learning, whereas CRF_2_ signaling contributes, in a context-dependent manner, to stress modulation and recovery (Koob and Schulkin, 2019). Within the hypothalamus, CRF inputs from the paraventricular nucleus and extended amygdala depolarize Hcrt neurons, promoting sustained firing and orexin release (Hu, 2016). This activation translates stress into heightened arousal and motivational urgency, biasing behavior toward drug seeking (Koob and Schulkin, 2019; Nestler, 2025).

At the circuit level, convergent CRF_1_ and OX1R signaling amplifies stress-induced reinstatement by stabilizing drug-cue associations and enhancing motivational salience. Synergistic engagement of shared downstream pathways supports persistent neuroadaptations that couple stress exposure to relapse vulnerability, consistent with negative-reinforcement models of addiction. Importantly, this interaction selectively implicates OX1R rather than orexin receptor 2 as a principal mediator of stress-induced reinstatement across multiple drug classes, highlighting a therapeutically relevant dissociation between relapse-related motivation and baseline arousal (James and Aston-Jones, 2022).

Counter-regulatory mechanisms also shape this balance. The nociceptin/orphanin FQ (N/OFQ) system, acting through G_i/o_ protein–coupled nociceptin opioid peptide (NOP) receptors, dampens CRF-driven excitation within the extended amygdala and related stress circuits, reduces anxiety- and depressive-like behavior, and attenuates stress-induced reinstatement of drug seeking (Ciccocioppo et al., 2019; Ubaldi et al., 2021). Although direct inhibition of orexin neurons by N/OFQ remains incompletely characterized, the N/OFQ–NOP system functionally opposes CRF–orexin signaling (Ubaldi et al., 2021).

Clinically, these interactions highlight the potential of targeting stress–orexin coupling to reduce relapse risk. Pharmacological inhibition of CRF_1_ receptors and selective OX1R antagonism each attenuate stress-induced reinstatement, and combined modulation of these systems may offer an additive benefit (James and Aston-Jones, 2022). Dysregulated CRF–orexin coupling is also implicated in anxiety, depression, and post-traumatic stress disorder, underscoring its relevance beyond addiction (Koob and Schulkin, 2019; Mahmoudi et al., 2020; RayatSanati et al., 2021; Volkow and Blanco, 2023). Future approaches should integrate stress, orexinergic, and anti-stress systems to improve relapse prevention and emotional regulation.

## 10 Cannabinoids and valence processing: A neurobiological perspective

Cannabinoids exert broad modulatory effects on neural circuits governing motivational valence and influence reinforcement indirectly rather than acting as primary reward signals. Δ^9^-Tetrahydrocannabinol (THC), the principal psychoactive component of *Cannabis sativa*, influences valuation and reinforcement largely through indirect modulation of mesolimbic dopamine pathways. By activating cannabinoid receptor type 1 (CB1) receptors on GABAergic and glutamatergic inputs to VTA dopamine neurons, THC produces net disinhibition and modest increases in dopamine release within the NAc and PFC, contributing to reinforcement and altered reward valuation (Volkow et al., 2017).

The endocannabinoid system (ECS)—comprising CB1 and CB2 receptors, endogenous ligands (anandamide and 2-arachidonoylglycerol), and metabolic enzymes—functions as a retrograde neuromodulatory system that fine-tunes excitatory–inhibitory balance and synaptic plasticity across corticolimbic networks (Katona and Freund, 2012). CB1 receptors, widely expressed in cortical, limbic, and striatal regions, regulate glutamate and GABA release and thereby shape reinforcement learning, cue salience, and affective regulation, while CB2 receptors contribute to neuroimmune and plasticity-related processes within striatal and limbic circuits (Jordan and Xi, 2019). Disruption of ECS signaling—through CB1 antagonism or genetic deletion—reduces drug-evoked dopamine release and cue reactivity, underscoring its role in modulating motivational processes (Wenzel and Cheer, 2018; Navarrete et al., 2022).

Developmental timing critically shapes the impact of cannabinoids on valence processing. Preclinical studies demonstrate that repeated THC exposure during adolescence—a sensitive period for frontostriatal maturation—induces long-lasting neuroadaptations in dopamine–glutamate interactions within the NAc and PFC, alters gene expression and epigenetic profiles, and enhances cue-driven control of behavior (Renard et al., 2017; Zuo et al., 2022; Murray et al., 2025). Notably, adolescent THC exposure increases Pavlovian-to-instrumental transfer, indicating exaggerated influence of reward-predictive cues on action selection and aligning cannabinoid effects with incentive-sensitization and habit-formation frameworks (Miller et al., 2019; Scherma et al., 2020; Orihuel et al., 2021).

Human neuroimaging studies provide partial convergence, revealing altered ventral striatal responses during reward anticipation, compensatory recruitment of prefrontal regions during decision-making, and disrupted functional connectivity among PFC, insula, and midbrain regions in chronic cannabis users (Zimmermann et al., 2019; Skumlien et al., 2022). Although effect sizes are heterogeneous, the overall pattern supports subtle but persistent perturbations in reward–control integration.

The ECS interacts with opioid, glutamatergic, GABAergic, orexinergic, and stress systems, shaping synaptic plasticity and motivational salience across interacting circuits (Mahler et al., 2014a; Sagheddu et al., 2015; Morales and Margolis, 2017; Wenzel and Cheer, 2018; Mahmoudi et al., 2020; RayatSanati et al., 2021). Cannabinoids can therefore be understood as modulators of motivational gain whose impact depends on developmental stage, genetic vulnerability, and network state. This perspective informs emerging translational strategies targeting ECS tone and plasticity to normalize cue-driven motivation while preserving its essential homeostatic functions (Sagheddu et al., 2015; Nestler, 2025).

## 11 Histaminergic system in valence and reward regulation: A modulator of drug reinforcement and behavioral flexibility

Addictive behaviors emerge from coordinated interactions among multiple neuromodulatory systems (Volkow and Morales, 2015). Within this context, the histaminergic system has gained attention as a regulator of motivational salience, arousal, and executive control, influencing impulsivity, behavioral flexibility, and relapse vulnerability. Histaminergic neurons originate exclusively from the tuberomammillary nucleus (TMN) of the posterior hypothalamus and project widely to corticolimbic and striatal regions, including the VTA, NAc, and PFC. Through this broad anatomical reach, histamine links internal state and arousal to reward evaluation and decision-making, integrating motivational drive with cognitive control (Panula and Nuutinen, 2013).

Histamine acts through four G protein–coupled receptors, conferring bidirectional and context-dependent modulation of neural circuits. Among these, the H_3_ receptor plays a particularly prominent role in addiction-relevant processes. Acting as a presynaptic auto- and heteroreceptor, H_3_ exerts inhibitory control over the release of histamine itself and other transmitters, including dopamine and glutamate, thereby regulating the gain and temporal precision of mesocorticolimbic signaling (Ferrada et al., 2009). Pharmacological blockade of H_3_ receptors reduces alcohol intake, attenuates relapse-like behavior, and diminishes psychostimulant- and opioid-seeking in multiple preclinical models, supporting a role for histaminergic modulation in restoring balance within motivational circuits (Munzar et al., 2004; Ligneau et al., 2007). In contrast, H_1_ and H_2_ receptor signaling supports wakefulness, attention, and aspects of cognitive flexibility, consistent with histamine’s broader role in adaptive behavioral regulation and executive control (Passani and Blandina, 2011).

Experimental evidence further indicates that histaminergic tone can buffer excessive dopaminergic activation. Enhancing histamine signaling attenuates stimulant-induced hyperlocomotion and sensitization in rodents, reinforcing the view that histamine constrains dopamine overactivation rather than simply inhibiting reward processing (Kubota et al., 2002; Takino et al., 2009). Within striatal and prefrontal regions, histaminergic projections modulate reward-based decision-making and inhibitory control, influencing the balance between goal-directed and habitual behavior and thereby shaping vulnerability to compulsive drug use (Torrealba et al., 2012).

Beyond reward, histamine forms a physiological bridge between arousal, circadian regulation, and motivational control. TMN histamine neurons are central to wake–sleep regulation, and disturbances in histaminergic tone—such as those arising from sleep disruption or circadian misalignment—have been proposed to alter reward sensitivity and stress responsivity, potentially increasing relapse risk, although direct causal evidence in humans remains limited (Panula, 2020). These interactions align with broader evidence for bidirectional coupling between reward circuits and circadian systems (Lewis et al., 2021).

At the circuit level, histamine and dopamine are tightly interconnected. High expression of H_3_ receptors in striatal territories enables histamine to exert layered, receptor-specific control over dopamine synthesis, release, and postsynaptic responsiveness (Ferrada et al., 2009; Panula and Nuutinen, 2013). This positions histamine as both a brake and a fine-tuner of dopamine-dependent reinforcement and cognitive processes, shaping not only acute reward responses but also relapse vulnerability and the persistence of addiction-related neuroadaptations (Ligneau et al., 2007; Ellenbroek, 2013; Munari et al., 2013; Nuutinen et al., 2016).

From a translational perspective, histaminergic mechanisms—particularly H_3_ receptor antagonism—represent a promising avenue to enhance cognitive control, reduce compulsivity, and lower relapse risk (Panula, 2020). The histaminergic system can thus be viewed as an integral component of brain networks regulating valence, arousal, and behavioral flexibility, operating in concert with other neuromodulatory systems.

## 12 Central ghrelin system: A modulator of addiction vulnerability

The central ghrelin system, classically associated with energy balance, has emerged as a state-dependent modulator of reward processing and addiction vulnerability. Ghrelin acts primarily via the growth hormone secretagogue receptor type 1A (GHS-R1A), which is widely expressed in reward-related regions, including the hypothalamus, VTA, NAc, and hippocampus (Abizaid et al., 2006; Perelló and Zigman, 2012). A distinctive feature of this system is the high constitutive activity of GHS-R1A, enabling modulation of dopaminergic excitability even in the absence of ligand and positioning ghrelin signaling at the interface between homeostatic and hedonic motivation (Jiang et al., 2006).

Preclinical evidence indicates that ghrelin enhances the motivational value of both natural and drug rewards. Central ghrelin administration increases operant responding for palatable food and augments alcohol-, cocaine-, and amphetamine-related reinforcement, in part by increasing VTA dopamine neuron firing and elevating NAc dopamine release (Dickson et al., 2011; Skibicka et al., 2013; Engel and Jerlhag, 2014). At the cellular level, ghrelin facilitates glutamatergic input to dopamine neurons and promotes NMDA receptor–dependent plasticity, biasing reinforcement learning toward heightened cue responsivity (Abizaid et al., 2006).

A critical feature of ghrelin’s action is its functional convergence with the endocannabinoid system. Ghrelin-driven excitation of VTA dopamine neurons requires CB1 receptor signaling to fully regulate excitatory afferents, establishing a synergistic mechanism through which metabolic state calibrates motivational salience (Kawahara et al., 2013; Edwards et al., 2025). Disruption of ECS signaling attenuates ghrelin-induced enhancement of food and drug reward, whereas inhibition of endocannabinoid degradation amplifies these effects (Davis et al., 2020; Edwards et al., 2025).

From a translational perspective, GHS-R1A antagonists and inverse agonists reduce alcohol intake, blunt drug-conditioned place preference, and attenuate relapse-like behavior in preclinical models (Jerlhag et al., 2009; Suchankova et al., 2013). These findings position ghrelin as a metabolic regulator of reward sensitivity whose dysregulation can bias behavior toward compulsive seeking. Targeting ghrelin–dopamine–ECS interactions may therefore offer a novel strategy for restoring motivational regulation in substance use disorders.

## 13 Galanin and reward processing: A modulator of addiction-related behaviors

Galanin is a widely expressed neuropeptide that modulates reward processing, stress responsivity, and neural plasticity, positioning it as an important regulator of addiction-related behaviors. Acting through three G protein–coupled receptors (GalR1, GalR2, and GalR3), galanin exerts receptor- and circuit-specific effects that often counterbalance excessive reinforcement and negative affect (Kuteeva et al., 2008; Mitsukawa et al., 2008). This receptor heterogeneity enables galanin to integrate motivational and stress-related signals across mesolimbic, limbic, and brainstem circuits.

Within reward pathways, galanin generally suppresses dopaminergic transmission and incentive salience, primarily through G_i/o_-coupled GalR1 and GalR3 signaling. GalR1 activation reduces neuronal excitability, inhibits dopamine neuron firing in the VTA, and decreases dopamine release in the NAc. Consistent with this mechanism, central or VTA-targeted galanin administration attenuates morphine-conditioned place preference, suppresses cocaine-induced hyperlocomotion, and reduces reinstatement of drug seeking (Zachariou et al., 2003; Narasimhaiah et al., 2009). Conversely, genetic disruption of galanin or GalR1 signaling enhances sensitivity to opioid and psychostimulant reward, supporting its role as a regulatory brake on excessive reinforcement (Kuteeva et al., 2005; Hawes et al., 2008).

In contrast, GalR2 signaling is more closely associated with stress adaptation and resilience. Coupling predominantly to G_q/11_ pathways, GalR2 can support context-dependent plasticity within prefrontal and amygdalar circuits, modulating anxiety-like behavior and withdrawal-related negative affect. GalR2-deficient mice exhibit altered stress responsivity and enhanced relapse-like behavior, suggesting that galanin contributes not only to dampening reward but also to buffering stress-driven relapse vulnerability (Kinney et al., 2002; Hawes et al., 2008; Hökfelt et al., 2018). At the molecular level, galanin reduces glutamatergic drive onto VTA dopamine neurons and modulates dopamine synthesis, further constraining reinforcement-related plasticity (Ericson and Ahlenius, 1999).

Galanin engages stress and arousal systems within the extended amygdala and locus coeruleus, where it modulates noradrenergic output and hypothalamic–pituitary–adrenal (HPA) axis activity. Chronic opioid exposure reduces galanin expression in the amygdala, whereas withdrawal upregulates GalR expression in noradrenergic nuclei, consistent with a role in mitigating hyperarousal and negative affect during abstinence (Weiss et al., 1998; Holmes et al., 2002). Human genetic studies linking GAL and GALR polymorphisms to opioid dependence further support a modulatory—though non-deterministic—role in addiction vulnerability (Levran et al., 2008).

Notably, galanin’s effects are substance- and circuit-specific. While it generally suppresses opioid and psychostimulant reinforcement, hypothalamic galanin activity can increase ethanol intake in genetically predisposed rodent strains, reflecting its dual role in feeding motivation and consummatory behavior (Rada et al., 2004; Karatayev et al., 2010). This complexity highlights the need for receptor- and circuit-selective approaches.

Collectively, galanin emerges as a key neuromodulator of valence regulation, integrating inhibitory control over reward with stress-buffering functions. Targeting galanin receptors—particularly GalR1 agonism or GalR2-biased modulation—may offer translational avenues to reduce relapse vulnerability, enhance stress resilience, and restore functional balance within reward–stress circuits (Cantero-García et al., 2022; Flores-Gómez et al., 2025).

## 14 Glial modulation of synaptic plasticity and reward signaling

Glial cells are now recognized as active regulators of synaptic plasticity within reward circuits, contributing to addiction-related neuroadaptations alongside neuronal mechanisms. Astrocytes in the NAc regulate extracellular glutamate via excitatory amino acid transporters, particularly excitatory amino acid transporter 2 (EAAT2), also known as glutamate transporter 1 (GLT-1), thereby shaping the excitability of medium spiny neurons and dopamine-dependent reinforcement. Chronic cocaine exposure and abstinence reduce astrocytic coverage and glutamate uptake, promoting spillover onto extrasynaptic receptors and facilitating maladaptive plasticity. Recent evidence further shows that microglia can phagocytose astrocytic processes during abstinence, exacerbating glutamate dysregulation and promoting relapse-like behavior (Scofield et al., 2016; Testen et al., 2025).

Microglia also influence reward-related plasticity through neuroimmune signaling. Drug exposure induces microglial activation and release of pro-inflammatory cytokines (e.g., TNF-α, IL-1β), which modify AMPA receptor trafficking and synaptic strength within mesocorticolimbic circuits, reinforcing drug-induced potentiation (Lacagnina et al., 2017; Skupio et al., 2020). Conversely, microglia-derived brain-derived neurotrophic factor (BDNF) supports experience-dependent plasticity, highlighting a dual role in adaptive and pathological learning (Parkhurst et al., 2013; Kopec et al., 2019).

Together, disrupted glutamate homeostasis, neuroinflammatory signaling, and altered trophic support position glial–neuronal interactions as central determinants of relapse vulnerability. Incorporating glial dynamics into addiction models highlights astrocytic and microglial pathways as translational targets, including restoration of EAAT2 function and modulation of neuroimmune tone (Kopec et al., 2019; Linker et al., 2019).

## 15 The subventricular tegmental nucleus: a distributed brainstem hub for dopaminergic and affective regulation

Recent work by Zichó and colleagues has identified the SVTg as a previously unrecognized GABAergic brainstem structure with potential relevance for reward processing (Zichó et al., 2025). Current experimental evidence supporting this role is primarily derived from this study, which demonstrated that SVTg neurons are activated by reward and reward-predicting cues, and that their optogenetic stimulation induces conditioned place preference, self-stimulation, and reward-seeking behavior. In addition, SVTg activity was shown to modulate dopaminergic output indirectly through inhibitory projections to the LHb, a key regulator of aversive and anti-reward signaling. These findings provide initial evidence that the SVTg contributes to positive valence encoding and motivational control.

Beyond these functional observations, emerging anatomical and systems-level frameworks suggest that the SVTg is embedded within a broader orbitofrontal cortex–SVTg–LHb–VTA axis, linking higher-order cortical inputs with subcortical circuits involved in valence evaluation and dopaminergic regulation (Chen and Hu, 2025; Grillner, 2025; Zichó et al., 2025). Convergent projections from prefrontal and limbic regions, including the orbitofrontal cortex, anterior cingulate cortex, and amygdala, position the SVTg at the interface between cognitive–affective processing and brainstem motivational systems (Rolls, 2017; Hu et al., 2019). Through its prominent inhibitory control over the LHb, the SVTg can suppress aversive signaling and facilitate reward-directed behavior, supporting its interpretation as an integrative node within distributed valence-processing networks rather than as an isolated reward center.

Electrophysiological data further indicate that SVTg activity tracks reward delivery and predictive cues, partially paralleling dopaminergic signaling while maintaining distinct temporal and functional dynamics (Zichó et al., 2025). Comparative analyses across vertebrate species suggest that SVTg–LHb–dopamine interactions are evolutionarily conserved, consistent with a broader role in coordinating approach–avoidance behavior (Grillner, 2025; Zichó et al., 2025). Within this framework, dysregulation of SVTg-centered circuitry may contribute to imbalances between reward and aversion, potentially biasing behavior toward excessive reward seeking or maladaptive avoidance.

However, this interpretation should be considered provisional. The identification of the SVTg as a reward-related structure is currently based on a limited number of studies, with direct evidence largely restricted to a single experimental report. Its role in addiction, its interactions with stress-related systems, and its functional relevance in humans remain to be established. Further work is required to determine whether the SVTg constitutes a central integrative hub in pathological conditions or represents one component within a broader, distributed regulatory network.

## 16 Reconceptualizing the reward system: balancing positive and negative valence

Dopamine has long been regarded as the central driver of reinforcement learning and addiction, shaping both theoretical models and pharmacological strategies (Volkow et al., 2017). However, converging evidence now indicates that while mesolimbic dopamine is necessary for many reward-related behaviors, it is neither sufficient nor singularly responsible for reinforcement or compulsivity. Reward processing instead emerges from a coordinated, valence-sensitive network in which positive and negative motivational signals are dynamically balanced across multiple neuromodulatory and cellular systems (França and Pompeia, 2023).

Within this framework, motivation and decision-making reflect the coordinated action of glutamatergic, GABAergic, peptidergic, monoaminergic, and neuromodulatory pathways, each contributing distinct regulatory functions to learning, salience attribution, and behavioral flexibility (Kim and Picciotto, 2023; Peng et al., 2025). Dopamine remains central but operates within a broader architecture that integrates arousal, stress, metabolic state, and executive control.

Recent work has further expanded this architecture by identifying brainstem nodes that may regulate motivational balance upstream of dopamine, most notably the SVTg, which has been proposed to modulate dopaminergic excitability via lateral habenula inhibition, potentially contributing to the regulation of approach–avoidance behavior (Grillner, 2025; Zichó et al., 2025). Dysfunction within this axis may bias motivational processing toward maladaptive behavioral states.

This framework is reflected across the systems described above. Orexin couples arousal to motivational drive and stress-induced relapse (Mahmoudi et al., 2020; RayatSanati et al., 2021); histaminergic signaling modulates attentional gain and inhibitory control (Di Ciano et al., 2022); the endocannabinoid system shapes plasticity and cue salience, particularly during neurodevelopmental windows (Sagheddu et al., 2015; Orihuel et al., 2021; Ramaekers et al., 2022; Cajiao-Manrique et al., 2023; Ferland et al., 2023; Lorenzetti et al., 2023; Ertl et al., 2024; MacNicol et al., 2025; Tseng and Molla, 2025). Beyond neurons, astrocytes and microglia actively regulate synaptic homeostasis and inflammatory tone, influencing relapse vulnerability and long-term circuit stability (Corkrum et al., 2020; Jiménez-González et al., 2022; da Silva et al., 2023; Serra et al., 2025).

These findings reframe addiction as a disorder of network-level dysregulation of motivational balance rather than a hyperdopaminergic state. This reconceptualization supports multi-targeted, circuit-informed interventions aimed at restoring adaptive regulation of reward and aversion, advancing psychiatry toward mechanistic, systems-level models of motivation and adaptive behavior.

## 17 Emerging therapeutic targets across neuromodulatory systems

**FIGURE 3:**
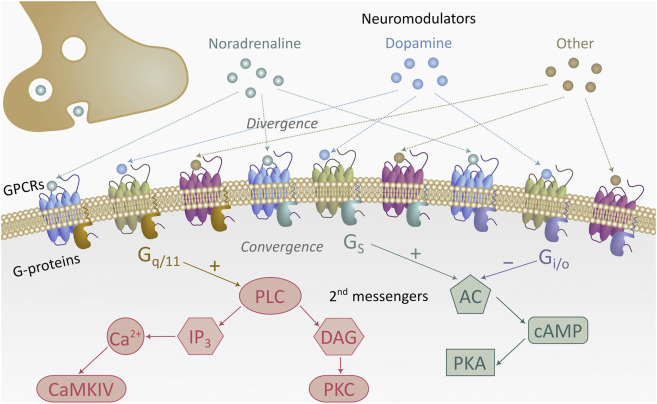
Neuromodulatory divergence and intracellular convergence in reward-related signaling pathways. Multiple neuromodulators, including noradrenaline, dopamine, and other neuromodulatory transmitters, engage distinct G-protein–coupled receptors (GPCRs) that couple to discrete heterotrimeric G-proteins (G_q/11_, G_s_, or G_i/o_). Despite the apparent divergence at the receptor level, these signaling cascades converge onto a limited set of canonical second messenger pathways that regulate neuronal excitability, synaptic plasticity, and transcriptional reprogramming in reward circuits. Activation of G_q/11_ stimulates phospholipase C (PLC), generating diacylglycerol (DAG) and inositol trisphosphate (IP_3_), which drive protein kinase C (PKC) and calcium/calmodulin-dependent kinase IV (CaMKIV) signaling. Receptors coupled to G_s_ activate adenylyl cyclase (AC), elevating cyclic AMP (cAMP) and engaging protein kinase A (PKA) to facilitate long-term synaptic potentiation. In contrast, G_i/o_ suppresses AC signaling and decreases cAMP, thereby counterbalancing excitatory drive. These transduction mechanisms allow neuromodulatory systems to broadcast diverse motivational signals while maintaining intracellular biochemical convergence, providing a mechanistic substrate for competitive interactions between reward, stress, and aversion during reinforcement learning and addiction vulnerability.

Neuromodulatory signaling operates through interacting G protein–coupled receptor networks whose effects are non-linear, context-dependent, and highly convergent (Figure 3). Rather than functioning as isolated switches, neuromodulators act through overlapping intracellular cascades whose integration determines excitability, plasticity, or inhibition at the circuit level (Treviño and Manjarrez, 2022). This architecture supports biological degeneracy, whereby distinct molecular pathways can yield similar functional outcomes, conferring resilience but also enabling maladaptive compensation in disease. These neuromodulatory systems operate across partially distinct temporal scales, from rapid phasic signaling to slower state-dependent modulation, which has important implications for circuit plasticity, behavioral flexibility, and therapeutic intervention (Table 3). Accordingly, addiction is increasingly understood as network-level dysregulation across interacting neuromodulatory pathways.

**TABLE 3:** Temporal dynamics of neuromodulators in reward processing.

Neuromodulator	Signaling timescale	Mode of action	Behavioral impact	Key regions
Dopamine (DA)	Phasic: milliseconds–seconds Tonic: seconds–minutes	Phasic bursts encode reward prediction errors, while tonic levels calibrate motivational tone through volume transmission	Supports reinforcement learning, effort allocation, and salience attribution	Ventral tegmental area (VTA), nucleus accumbens (NAc), dorsal striatum (Schultz, 2016; Zimmerman and Grace, 2016)
Serotonin (5-HT)	Tonic signaling over minutes–hours	Acts via volume transmission; modulates mood, patience, and sensitivity to punishment	Regulates impulsivity and aversive learning, facilitating inhibitory control	Raphe nuclei, prefrontal cortex (PFC), striatum (Cools et al., 2011; Volkow and Boyle, 2018)
Acetylcholine (ACh)	Tonic or cue-locked bursts (sub-second–minutes)	Cholinergic inputs enhance sensory salience and cortical plasticity; they regulate DA neuron burst firing	Promotes attention, cue detection, and behavioral flexibility	Pedunculopontine and laterodorsal tegmental nuclei (PPT/LDT), basal forebrain, VTA (Mahler et al., 2014b; Morales and Margolis, 2017)
Orexin (hypocretin)	State-dependent; phasic or sustained activity aligned with arousal and circadian cycles	Excitatory neuropeptides that couple arousal with motivational drive	Drive drug-seeking reinstatement and stress-induced craving	Lateral hypothalamus (LH), VTA, NAc, locus coeruleus (Mahler et al., 2014a; James and Aston-Jones, 2022)
Cannabinoids (endogenous)	Tonic or event-triggered release (seconds–minutes)	Retrograde signaling via CB1 receptors inhibits presynaptic glutamate and GABA release	Modulate synaptic plasticity, hedonic tone, and habit formation	Cortex, hippocampus, striatum, VTA (Katona and Freund, 2012; Volkow et al., 2017)

This perspective has direct translational implications. Therapeutic development is shifting from dopamine-centric strategies toward selective recalibration of distributed motivational, arousal, and control systems (Jovanoski et al., 2023). Among these, the orexin/hypocretin system has emerged as a key target linking arousal to cue-driven motivation. Dual orexin receptor antagonists, including suvorexant and lemborexant, reduce cue- and stress-induced drug seeking in preclinical models and are being explored clinically for relapse prevention, although efficacy across substance classes remains under evaluation (Mahler et al., 2014a; James and Aston-Jones, 2022).

The endocannabinoid system offers additional opportunities. While first-generation CB1 antagonists were limited by psychiatric adverse effects, biased or peripherally restricted compounds aim to modulate mesolimbic signaling with improved tolerability (Calleja-Conde et al., 2022). Neurometabolic agents such as GLP-1 receptor agonists (e.g., semaglutide) have shown promise in reducing craving for alcohol, nicotine, and stimulants, likely by integrating satiety and motivational signals at the level of midbrain dopamine neurons (Thomsen et al., 2019; Eren-Yazicioglu et al., 2021). The histaminergic system has also gained translational relevance: H_3_ receptor antagonists enhance cortico-striatal control, reduce impulsivity, and attenuate relapse-like behavior in preclinical models (Bahi et al., 2013; Sadek et al., 2016; Rapanelli, 2017).

Finally, the recent identification of the SVTg as a brainstem regulator of lateral habenula–dopamine interactions highlights possible circuit-level intervention points, with potential relevance for both addiction and mood disorders (Grillner, 2025; Zichó et al., 2025). Together, these approaches exemplify a shift toward multimodal, circuit-informed interventions targeting interacting neuromodulatory systems, advancing treatment beyond linear neurotransmitter models toward systems-level precision psychiatry.

## 18 Future directions and testable hypotheses

Advancing this framework requires a reorientation of experimental and translational priorities in addiction research toward explicitly testable, multi-scale models of motivational control. A central hypothesis emerging from recent work is that the SVTg may function as a state-dependent regulator of motivational dynamics by potentially exerting inhibitory control over the lateral habenula and, indirectly, midbrain dopamine systems (Grillner, 2025; Zichó et al., 2025). Cell-type- and projection-specific manipulation of SVTg–LHb pathways could help test whether dysregulation of this axis biases reinforcement learning toward compulsive reward seeking despite aversive outcomes (Jovanoski et al., 2023). A critical prediction is that selective modulation of this pathway will bidirectionally alter approach–avoidance behavior and relapse-like responses even in the absence of direct dopaminergic manipulation; failure to observe such partial dissociation would argue against a primary role of this axis in governing motivational control.

At the cellular level, a second priority is to map neuromodulatory and glial plasticity at single-cell resolution across addiction-relevant hubs, including the VTA, NAc, hypothalamus, and extended amygdala. Single-cell transcriptomic and epigenomic profiling during acute versus chronic drug exposure may reveal receptor reconfiguration, co-transmitter shifts, and astrocyte–microglia adaptations that stabilize maladaptive learning and relapse vulnerability (Lacagnina et al., 2017; Nestler, 2025). Within this framework, a key prediction is that restoration of astrocytic glutamate homeostasis—particularly through normalization of EAAT2/GLT-1 function—or targeted modulation of microglial activation states should reverse pathological synaptic plasticity and reduce relapse-like behavior independently of direct neuronal receptor manipulation. Failure to observe convergent synaptic and behavioral recovery would argue against a causal role for glial mechanisms in sustaining addictive phenotypes.

At the integrative systems level, the model further predicts that metabolic and interoceptive neuromodulators contribute to reward regulation by interacting with mesocorticolimbic and habenular–brainstem circuits. In particular, GLP-1 signaling is expected to modulate motivational salience and drug seeking through distributed effects on dopaminergic and non-dopaminergic pathways (Klausen et al., 2022). Pharmacological activation of GLP-1 receptors should reduce drug intake and cue-induced craving in a state-dependent manner, interacting with satiety, stress, and interoceptive signals. A critical test of this hypothesis is that circuit-specific manipulation of nucleus tractus solitarius-derived GLP-1 projections to the VTA, nucleus accumbens, and habenula will differentially affect reward valuation, aversion, and relapse propensity. Moreover, coordinated modulation of metabolic and arousal systems—such as combined GLP-1 receptor activation and orexin receptor antagonism—is predicted to produce non-additive or synergistic effects on motivational behavior; strictly additive or absent interactions would argue against meaningful cross-system integration.

In humans, longitudinal neuroimaging combined with glial and inflammatory biomarkers offers a translational bridge. PET imaging of microglial activation, MR spectroscopy, and peripheral inflammatory markers could be paired with task-based fMRI of mesocorticolimbic and habenular–brainstem circuits to track motivational dysregulation and relapse risk over time. Such approaches may help identify biomarkers that reflect the balance between reward, anti-reward, and homeostatic systems.

Finally, the cerebellum has emerged as a candidate contributor to reward and valence processing, given its connectivity with striatal, midbrain, and prefrontal circuits and its sensitivity to repeated drug exposure (Wang and Lan, 2023; Darvishzadeh-Mahani et al., 2025). The framework predicts that cerebellar perturbation will modulate reinforcement learning, prediction error signaling, or habit formation in a context-dependent manner. Systematic investigation of cerebellar contributions thus represents a testable extension of distributed reward models.

Together, these directions support multi-scale, testable models of addiction spanning molecular, cellular, and circuit levels, enabling precision, circuit-informed interventions.

## 19 Discussion

The evidence reviewed here supports a shift from a linear dopamine-centric model to a network model in which reward, aversion, and behavioral control emerge from interactions among partially dissociable but strongly coupled systems, including dopaminergic, peptidergic, endocannabinoid, histaminergic, metabolic, glial, and cortical–brainstem regulatory processes (Koob and Volkow, 2016; Lüscher and Janak, 2021; Volkow and Blanco, 2023; Nestler, 2025). In this view, dopamine remains indispensable for many forms of reinforcement learning and incentive motivation, but its effects are gated, amplified, constrained, or reweighted by parallel systems that encode stress, arousal, interoception, inflammatory tone, and executive control (Kahnt and Schoenbaum, 2025; Robinson and Berridge, 2025). Accordingly, addiction is better conceptualized not as a hyperdopaminergic disorder *per se*, but as a failure of coordinated network regulation in which the balance between approach-promoting and avoidance-promoting processes becomes progressively biased toward compulsive drug seeking. This formulation accommodates a wider range of findings than classic dopamine models, including persistent drug seeking under punishment, stress-induced relapse, blunted responsiveness to natural rewards, and the prominent role of non-dopaminergic interventions in modulating addiction-like behavior (Koob and Volkow, 2016; Kim and Picciotto, 2023; Valentino et al., 2024).

A central implication of this network perspective is that neuromodulatory systems should not be treated as parallel “add-ons” to dopamine, but as interacting control layers with partially distinct computational roles. Orexin links arousal and internal state to motivational vigor and cue responsiveness; dynorphin and CRF bias the system toward negative affect and negative reinforcement; endocannabinoids regulate synaptic gain and plasticity; histamine modulates attentional allocation and behavioral flexibility; ghrelin and GLP-1 embed reward processing within metabolic and interoceptive state; and glial mechanisms shape the permissive inflammatory and homeostatic environment in which these signaling systems operate (Koob, 2021; Klausen et al., 2022; Valentino et al., 2024). These systems converge on overlapping hubs—including the ventral tegmental area, nucleus accumbens, prefrontal cortex, extended amygdala, lateral habenula, and now potentially the SVTg—where they can alter both immediate motivational output and the longer-term plasticity that stabilizes addictive behavior (Lüscher and Janak, 2021; Grillner, 2025; Zichó et al., 2025). Thus, the most useful organizing principle is not transmitter identity alone, but how each system contributes to network functions such as salience allocation, reward prediction, stress recruitment, action selection, and behavioral flexibility.

Within this framework, the SVTg–lateral habenula axis represents one of the most provocative recent additions because it offers a potential upstream mechanism for regulating dopaminergic and aversion-related circuitry at the brainstem level (Grillner, 2025; Zichó et al., 2025). The available data suggest that the SVTg is a GABAergic brainstem structure with reward-related properties that has been shown to inhibit the lateral habenula, and to promote place preference, reduce anxiety-like behavior, and be engaged by reward and reward-predicting cues in rodents (Zichó et al., 2025). Conceptually, this positions the SVTg as a candidate integrative node through which cortical, limbic, and midbrain signals may bias motivational behavior toward approach or avoidance. However, this interpretation remains preliminary. First, the evidence is overwhelmingly preclinical and heavily dependent on optogenetic, viral-tracing, and cell type-specific manipulations performed under highly controlled conditions. Such methods are powerful for causal dissection but may exaggerate circuit specificity relative to physiological dynamics. Second, claims of translational conservation remain inferential: although anatomical markers suggest homologous structures in rat, macaque, and human brainstem, there is currently no direct functional evidence that the human SVTg plays an analogous role in reward regulation, relapse, or affective disorders (Grillner, 2025; Zichó et al., 2025). Third, the degree to which this circuit contributes to addiction, rather than more general reward learning or anxiety regulation, remains unresolved. The SVTg therefore should be regarded as a compelling hypothesis-generating discovery rather than an established clinical target.

More broadly, the literature across neuromodulatory systems reveals a recurring tension between mechanistic richness in animal models and limited translational traction in humans. Orexin is a clear example. Preclinical evidence strongly implicates orexin in cue-induced reinstatement, high-effort reward seeking, and stress-related relapse, particularly through OX1R-mediated mechanisms. Yet this mechanistic plausibility has not yet translated into robust clinical evidence that orexin antagonism substantially modifies addictive behavior in humans. The relatively modest and heterogeneous effects observed clinically, together with the complex phenotype of narcolepsy, argue against simplistic interpretations in which orexin is treated as a singular “relapse transmitter” (Koob, 2021). Similar caution applies to histamine: the preclinical data support a role in attentional gain, inhibitory control, and modulation of corticostriatal function, but direct evidence for clinically meaningful effects of histaminergic manipulation in addiction remains sparse. In both cases, the animal literature likely captures genuine mechanisms, but the human relevance of these mechanisms may depend on disease stage, substance class, sleep state, comorbidity, or baseline circuit dysfunction—variables that are often minimized in preclinical designs but are central in clinical populations.

The endocannabinoid and metabolic systems further illustrate why an integrated network model is preferable to a transmitter-centered one. The endocannabinoid system is well positioned to modulate motivational salience because it regulates glutamatergic and GABAergic transmission across corticolimbic circuits and is highly sensitive to developmental timing, environmental context, and prior drug exposure. However, the translational literature remains complicated by major sources of heterogeneity, including cannabis potency, route of administration, polysubstance use, age at exposure, and premorbid psychiatric vulnerability. Adolescent exposure studies are especially informative mechanistically, but their clinical interpretation is limited because they cannot fully disentangle causal drug effects from pre-existing liability. Similarly, the literature on GLP-1 is promising but still immature. GLP-1 receptor agonists reduce alcohol and drug intake in multiple preclinical models, and early human studies suggest potential effects on craving and abstinence, yet the mechanisms remain uncertain and the clinical signal is still modest and incomplete (Klausen et al., 2022). Importantly, the available review evidence emphasizes that GLP-1 effects may not be explained solely by reduced reward, but may also involve satiety, aversion, stress-linked negative feedback, and habenula-related mechanisms; moreover, proposed dopamine transporter effects may be species dependent and not reproduced in humans (Klausen et al., 2022). These issues are not peripheral details: they mean that GLP-1 should currently be considered a state-dependent network modulator with translational potential, not a validated anti-addiction mechanism.

Glial mechanisms are particularly important for any cohesive network model because they introduce slower, permissive, and homeostatic forms of regulation that cannot be captured by neuron-only accounts. Astrocytic glutamate transport, microglial activation, cytokine signaling, and trophic modulation all have the capacity to reshape reward circuitry by altering extracellular glutamate tone, synaptic plasticity, and inflammatory set points (Lacagnina et al., 2017; Skupio et al., 2020; Nestler, 2025). In conceptual terms, glia may determine whether neuromodulatory perturbations remain transient or become stabilized into relapse-prone states. Yet here too the translational gap is substantial. Most evidence comes from rodent models using experimental manipulations that are difficult to map directly onto human addiction, and the available human markers—PET ligands for neuroinflammation, peripheral inflammatory markers, or magnetic resonance spectroscopy measures—remain indirect, nonspecific, and variably reproducible. The field therefore faces a methodological asymmetry: glial processes are increasingly central to mechanistic theories, but they are still difficult to quantify in living humans with enough specificity to guide intervention.

At the computational level, the review also highlights a mismatch between the complexity of the biology and the simplicity of many prevailing models. Classical reward-prediction-error accounts remain highly useful, but they cannot fully explain punishment-resistant drug seeking, prolonged negative reinforcement, or learning about reward features that go beyond scalar value (Kahnt and Schoenbaum, 2025). Likewise, polygenic risk approaches capture only a modest fraction of addiction vulnerability and remain difficult to interpret mechanistically in the absence of richer intermediate phenotypes (Lüscher and Janak, 2021; Volkow and Blanco, 2023). What is needed are models that explicitly integrate multiple control layers: fast dopaminergic prediction signals, slower stress and anti-reward processes, even slower glial and epigenetic stabilization, and top-down cortical modulation of action selection. Such models are conceptually attractive, but they are currently underdetermined by the data because few studies simultaneously measure behavior, circuit dynamics, and molecular or glial states within the same individuals or across comparable preclinical and clinical paradigms.

The translational consequences of this analysis are substantial. First, the limited efficacy of many candidate treatments should not be interpreted simply as evidence that the underlying biology is wrong. In several cases, negative or weak clinical findings may reflect problems of target engagement, inadequate patient stratification, endpoint selection, or the fact that a mechanism is only relevant in a subset of individuals or at a specific stage of the addiction cycle. The literature on CRF1 antagonists is instructive: despite strong preclinical rationale and evidence of endocrine target engagement, human laboratory studies have yielded largely negative or equivocal behavioral results, and no convincing clinical efficacy has yet emerged for major stress-related psychiatric disorders or addiction (Koob, 2021). This does not necessarily invalidate the broader stress-framework, but it does show that mechanistic plausibility alone is insufficient for therapeutic translation. Second, many interventions are still evaluated in diagnostically broad populations without biomarkers that identify the circuit dysfunction they are intended to modify. This is a major obstacle for neuromodulatory therapies such as rTMS or deep brain stimulation, whose effects are likely to depend critically on whether the targeted network is actually dysregulated in a given patient (Lüscher and Janak, 2021; Valentino et al., 2024). Third, implementation constraints matter. Even compelling biological advances may fail clinically if they are difficult to deliver chronically, poorly tolerated, too expensive, or not adaptable to diverse health systems and comorbidity profiles.

These translational limitations also underscore important differences between preclinical and clinical evidence. Animal models excel at causal specificity but often simplify the phenotype, typically isolating one substance, one stressor, one behavioral endpoint, and one developmental window. Human addiction, by contrast, is shaped by polysubstance use, social stress, psychiatric comorbidity, developmental heterogeneity, sex differences, sleep disturbance, and structural determinants of health (Volkow and Blanco, 2023; Valentino et al., 2024). This difference in dimensionality has methodological consequences. A manipulation that robustly reduces reinstatement in rodents may correspond to only a small effect on relapse risk in humans because the human phenotype is sustained by multiple partially redundant processes. Conversely, clinically important variables such as access to treatment, adherence, socioeconomic instability, or trauma exposure are seldom modeled adequately in basic studies. Progress will therefore require tighter reverse translation, more ecologically valid behavioral phenotyping, and multimodal longitudinal studies that can bridge circuit-level hypotheses with real-world clinical trajectories.

An additional unifying but underdeveloped dimension is time. The systems reviewed in this article operate on partially distinct temporal scales: dopamine on phasic and tonic timescales, orexin and stress systems across arousal states, endocannabinoid and glial processes across slower homeostatic and inflammatory windows, and metabolic signals across feeding, circadian, and endocrine rhythms. Most addiction studies, however, sample only one of these temporal layers. As a result, the field still lacks robust models of how short-timescale incentive signaling becomes embedded within longer-timescale stress and homeostatic dysregulation. This limitation is not merely theoretical. It may explain why acute laboratory measures often correlate weakly with long-term clinical outcomes and why interventions that change short-term craving do not necessarily alter relapse trajectories. Future work should therefore treat temporal organization as a core feature of addiction neurobiology rather than as a secondary consideration.

Taken together, the literature supports a cohesive network model in which addiction reflects a progressive failure of flexible valence regulation across interacting neuromodulatory, glial, metabolic, and cortical–brainstem systems. The value of this model lies not in replacing one master transmitter with several others, but in explaining how multiple systems with distinct functions and timescales converge on shared behavioral phenotypes: exaggerated cue-driven approach, diminished valuation of natural rewards, impaired avoidance learning, stress-triggered relapse, and reduced executive flexibility. At the same time, the review also makes clear that the empirical basis for this model is uneven. Some nodes and mechanisms are well supported in preclinical work but weakly validated in humans; some translational candidates are biologically plausible but clinically unproven; and several of the most integrative ideas still outpace the available methods. A critical next step is therefore not simply to expand the list of implicated systems, but to test explicit interaction models across species, scales, and clinical contexts. Only then can systems-level theories of reward and addiction support precision, circuit-informed interventions that are both mechanistically sound and clinically meaningful.
